# The use of Traditional Medicine by Ghanaians in Canada

**DOI:** 10.1186/1472-6882-8-30

**Published:** 2008-06-16

**Authors:** Kofi B Barimah, Edwin R van Teijlingen

**Affiliations:** 1 Faculty of Public Health and Allied Sciences, Catholic University College of Ghana-Fiapre, P. O. Box 363, Sunyani, Brong Ahafo, Ghana; 2 Department of Public Health, University of Aberdeen, School of Medicine & Dentistry, Aberdeen, AB25 2ZD, Scotland, UK

## Abstract

**Background:**

Research into health and health-care seeking behaviour amongst immigrant populations suggests that culturally-based behaviours change over time towards those prevalent in the host culture. Such acculturation of immigrant groups occurs as part of the interaction of immigrants with mainstream culture. This study examined the acculturation of Ghanaian immigrants in Greater Toronto Area (Canada) focusing particularly on attitudes towards and usage of Ghanaian traditional medicine (TRM).

**Methods:**

The study used both quantitative and qualitative methods. Structured questionnaire interviews were conducted with a sample of Ghanaians in active collaboration with the Ghanaian-Canadian Association in the Greater Toronto Area (GTA). A total of 512 questionnaire interviews were conducted. In addition, three focus groups of nine participants each were conducted with a sub-sample of Ghanaians in Canada.

**Results:**

Both the questionnaire and the focus groups indicated that nearly 73% of the Ghanaian immigrants in Canada have a positive attitude toward Ghanaian TRM. This is in comparison with less than 30% who have changed their attitude for various reasons. Some of the attraction of TRM lies in its holistic origin. Ghanaians in the GTA have been pursuing 'integration' and 'assimilation' in their acculturation in Canada. Some have given up or modified some of their attitudes and opinions toward TRM to embrace the 'modern' or 'civilized' way of living.

**Conclusion:**

There is the need for health care providers and other stakeholders to be aware of the influence of religion on African immigrants during their acculturation process. Although modernity is said to be founded on the 'ruthless undermining of tradition', there is no evidence to suggest that Ghanaian traditional religion has been undermined to such an extent that there is a major change in attitudes towards TRM.

## Background

Scholars have tried to explain why people change their opinions, views or perceptions about certain issues such as traditional medicine (TRM) as they migrate from their homeland to a different socio-economic and cultural environment. To what extent does 'modern life' affect the opinions, perceptions, attitudes and views of Africans in particular and immigrants in general as they move from their native countries to industrialised ones? Some have argued that when immigrants from Africa start adopting 'modern life', the beliefs in and the practice of TRM will become a thing of the past [1]. More generally, Herberg noted "a great many issues are created in a host country when people who have been born and brought up in other parts of the world enter, settle and integrate into that host society" [2]. Many of the 80,000 Ghanaians in Canada settled in and around Toronto,

"Such a transplantation of people from a socio-economic background characterized by traditional values, loyalties, informality, and low technological development to the advanced industrial setting of Toronto, poses challenges for both the refugee and the prevailing institutions [3]."

In the eyes of some, the solution lies in the immigrants' adaptation of the dominant norms and values of Canadian society, not only for the well-being of the individual immigrant, but also for the sake of political good-will amongst Canadians.

"Any clash which may arise between the demands of their traditional background and the universalistic norms in the Toronto social context, have implications for the acculturation of the Ghanaian refugee. The successful adaptation of immigrants to their new social environment is crucial, not only because of the problems that maladjustment can create, but also because evidence suggests that support for immigrant resettlement among Canadians is contingent upon it [3]."

Among other issues, it is not clear to what extent Ghanaian immigrants in the GTA have changed their attitudes to and opinions on TRM as a result of their stay in Canada where for example, the socio-economic and political structure, cultural expectations and health-care systems are different from that of Ghana. Studies have concentrated on other issues such as residential behaviour and ethnic community formation [4], being a refugee [3], development of women [5], and spatial aspects of labour market activity [6], but our study is the first one to focus on perceptions and attitudes of Ghanaian immigrants in Canada towards TRM. Therefore, this study gathered the opinions of a sample of the Ghanaians in the GTA, in order to determine to what extent they have changed their attitudes towards TRM as a result of their staying in Canada.

The terms 'immigrants' and 'refugees' have been used interchangeably in this paper because studies among Ghanaians in Canada point to the fact that there is really no significant difference between the experiences of these different patterns of migration during their acculturation process in Canada [5]. Indeed, some researchers have raised some doubts about the genuineness of most of these 'refugee' claimants, and consequently, we did not find it necessary to differentiate between 'immigrants' and 'refugees' [5]. The issue of 'illegal refugees' has been elucidated elsewhere in this paper (see section on acculturation and TRM). Thus, it is conceivable that a majority of those who claim to be 'refugees' (forced migration) during the study period may have actually entered Canada through routine migration or conventional channels.

### Traditional medicine

TRM can be studied for several perspectives, including anthropology, botany, sociology of religion and, more recently, medical sciences [7]. Therefore, it is important to define TRM first. In 1976, experts at the World Health Organization's (WHO) meeting in Brazzaville in Africa defined TRM as follows:

"The sum total of all the knowledge and practices, whether explicable or not, used in diagnosis, prevention and elimination of physical, mental and social imbalance and relying exclusively on practical experience and observation handed down from generation to generation, whether verbally or in writing...Traditional medicine might also be considered to be the sum total of practices, measures, ingredients and procedures of all kinds, whether material or not, which from time immemorial had enabled the African to guard against diseases, to alleviate his sufferings and to cure himself...Traditional medicine might also be considered as a solid amalgamation of dynamic medical know-how and ancestral medicine [8]."

### Acculturation

Although this paper was based on a theoretical framework of 'acculturation', the concept of 'inculturation' emerged as the main link between evangelical Christianity and acculturation. More recently, acculturation has also been defined as the:

"Movement from a situation in which one is a member of a group having its own well-internalized culture to a situation in which the individual is a non-member of a different identity group [9]."

Acculturation has also been criticized on the grounds that it is too aggressive, and does not convey the aspects of dialogue and mutual fusion essential for the meeting of two cultures [10]. According to Owoahene-Acheampong [11], the concept of 'inculturation' has influenced theologians in their analysis of what happens when two or more cultures meet. From an anthropological point of view, the process of inculturation is said to have the characteristics of acculturation [12].

### Acculturation and Traditional Medicine

Although descriptive studies point to a 'healthy immigrant' effect, suggesting that immigrants, especially recent ones, are less likely than the Canadian-born population to have chronic conditions or disabilities [13], the longer immigrant populations stay in Canada the more their health converges with that of other Canadians [14]. Length of stay in Canada also affects the extent to which a new immigrant may adapt to the wider Canadian culture [15]. Within the first 18 months, the difficulties experienced relate to variables pertaining to reception given by Canadians in terms, for example, availability of jobs and housing. Longer-term acculturation problems include, for example continued loss of social status, continued inability to speak English or French (the official languages) and differential rates of acculturation within families [15]. However, in a study on acculturation and health behaviours among Latino immigrants to the US, it was indicated that:

"Increased years of residence in the United States had the predictable impact of increased competence in English and increased use of English, but had differing impact by country of origin on the cultural orientation of the respondents' environment and the ethnic identification [16]."

According to Hiebert and colleagues, after a period of approximately ten years in a new country, immigrants report a distinctive sense of familiarity with their new country, but those from non-European backgrounds (such as Ghanaians) tend to experience more difficulties which may be attributed to cultural and language differences between their native country and Canada [17]. At the same time there is evidence among some immigrant groups that the longer they stay in their adopted the more they focus on the original culture and its values. For example, it was found that as Koreans' length of time in Canada increased, they tended to identify more with their Korean identity [18]. This stems from the fact that as some Koreans entered the university setting, where ethnic identity was encouraged and their physical features were acknowledged as a unique characteristic of Asians. Consequently, Kim and Berry have suggested that the higher the Koreans' level of education, the more they became aware of conflict, prejudice, and hardships, which then reinforce their desire for their cultural values [18]. However, these might be idealized versions of Korean identity.

Kim and Berry surveyed the acculturation attitudes of highly educated Koreans in the Greater Toronto Area (GTA) and concluded that there are several variables that are significant predictors of integration and assimilation respectively [18]. For the former, reading more Canadian newspapers, preferring to be interviewed in English, coming directly to Canada, and participating more in Canadian organizations were the main predictors. The predictors of assimilation were: reading fewer Korean newspapers, viewing fewer Korean television programs, having more Canadian and fewer Korean friends, hoping their children maintain less of Korean language, and migrating independently to Canada. The degree of participation in the Canadian culture was a reliable predictor of both assimilation and separation, while less education was found to be the predictor of marginalization and integration among the Koreans in the GTA [18].

According to the Canadian Task Force on Mental Health of Immigrants and Refugees (CTFMHIR), age at the time of migration affects the adaptation process. Young adults, adolescents, and older immigrants (seniors) find it difficult to adapt for different reasons. [19]. Developmental tasks and maturational identity crisis make it difficult for the young to adapt while the seniors or older persons are vulnerable to the stress of migration. Moreover, non-White immigrants often had lower educational achievements than other Canadians, for example:

"Immigrants were significantly overrepresented in the 'no schooling' and 'completed bachelor's education or more' categories, and non-immigrants were overrepresented in the 'some high school' category. On the whole then, immigrants' educational attainment is polarized and non-European well-established immigrants are less likely to be very poorly educated [13]."

Socio-economic status plays an important role in the adaptation process. Some studies have demonstrated that a majority of immigrants are at the lower end of the socio-economic ladder leading to problems in acculturation [13,20]. In a study on the illness experience, meaning and help-seeking among Chinese immigrants in Canada, Lee and his colleagues noted that underemployment was prominent among perceived causes of illness experience [20].

According to Opoku-Dapaah, a majority of Ghanaian immigrants (51.4%) were not happy with the acculturation process in Canada and this stemmed from "loss of pre-migration socio-economic status, discrimination and the lengthy immigration process [3]." In a study to examine how Ghanaian immigrants constructed their social world within Canada (the first of its kind among Ghanaian immigrants abroad), Opoku-Dapaah's impression:

"Of the behavioral practices of Ghanaian refugees was that they involve a considerable degree of cultural retention, with some adjustment or modification made based on circumstantial demands, or when deemed crucial for meeting personal motives. The extent of such retention and modification of behavior varies from individual to individual and remains contingent on the particular situation [3]."

It appears that being a refugee must relate to the expectation of being in Canada for a short period due to involuntary migration. This must lead to lower levels of acculturation. Based on the above observation, it is therefore, not surprising that illegal refugees do not readily disclose their personal information to outsiders. For instance, it has been observed among Ghanaian women that:

"Stories about events that have shaped the experiences and identities of immigrant women changed as the context changed. For instance, the pressures of immigration policy elicited stories that created a specific identity to suit an immigrant's claims while informally the same person presented an identity different from what is officially known. Sometimes, during off-tape conversations, some interviewees explained how they had to claim some status in order to match their sponsors' stories to the immigration officials. Although these tensions suggested how life stories may be narrated differently according to context and circumstance, the real problem lay with how these contextual narratives could be reconciled in terms of writing a meaningful historical account [5]."

Despite these concerns, the socio-economic status of the immigrant is also an important factor in the acculturation process. Immigrants living in Canada less than 10 years are significantly overrepresented in the lowest and lower middle income groups, and are significantly underrepresented in the highest one [13].

## Methods

This study uses a mixed-methods approach, consisting of quantitative questionnaires and qualitative focus groups with Ghanaians living in GTA. Individual social science research methods have their own methodological and philosophical limitations [19,20]. Using qualitative and quantitative methods in combination helps to enlarge on data generated by individual methods and allows for triangulation of findings Participants were selected from members of the 70 social and cultural associations in the GTA. In consultation with the executive members of these associations, all 4,045 names of the members of the associations were obtained through stratified sampling and a probability sampling method was used to select 600 respondents for the questionnaire survey. This method assures that the sample is fairly representative of the membership of the associations of Ghanaian immigrants in the GTA in terms of age, sex, ethnic background, income level, educational level, number of years in Canada, religion, place of birth and religion.

Other researchers have also used data from associations to select their participants for their studies, e.g. Opoku-Dapaah reported that some 70% of respondents participated in such groups [3]. One problem with these associations is that they are voluntary organisations which do not necessarily have the best kept record systems for researchers to access, for example: "some did not have files and those that did sometimes had patchy records [5]." Our study encountered similar problems as far as registration of members was concerned.

The questionnaire was piloted among Ghanaians in London, England to avoid contamination of the target population in GTA [21]. The questionnaire data was entered on an electronic database and analysed using SPSS.

Focus groups were designed for a sub-sample of the questionnaire respondents. Focus groups are said to be better able to "reveal the intensity of feelings, thus facilitating comparisons among different positions [33]." Focus groups were facilitated by the first author. Participants were assured anonymity and confidentiality, there were asked permission to tape record the focus group and published selected quoted. Quotes used in this paper offer some details about the focus group participant, but no names or details of their residency. The first two focus groups were selected using a random sampling of 18 out of those respondents who indicated during the survey that they were also available for focus group discussions. Purposive sampling was used to select nine more for the third focus group discussion. The qualitative data analysis took place through reading and re-reading the transcripts separately by both authors and themes were compared for consistency. A thematic approach was used, i.e. categories (or themes or codes) are developed from the dataset [34].

## Results

The demographic details of the 512 (85.3%) participants are listed in Table 1. Three focus groups were conducted with a total of 27 participants (nine in each group). The main results of the questionnaire survey are outlined in Table 2.

**Table 1:** Demographic information (n = 512)

	Frequency	%
**Place of Birth**		
Ghana	450	87.9
Canada	62	12.1
**Gender**		
Male	285	55.7
Female	227	44.3
**Marital Status**		
Married	260	50.8
Single	252	49.2
**Age**		
Less than 20 years	45	8.8
20–39 years	206	40.2
40–59 years	246	48.0
60–79 years	15	2.9
**Educational Level**		
No Schooling	7	1.4
Primary	7	1.4
Middle	231	45.1
Secondary/Technical	195	38.1
University	72	14.1
**Religion**		
Christian	317	61.9
Traditionalist	94	18.4
Free Thinker	52	10.2
Muslim	49	9.6
**Ethnic Group**		
Akan	225	43.9
Ga	72	14.1
Ewe	66	12.9
Hausa	53	10.4
Frafra	45	8.8
Mamprusi	35	6.8
Dagomba	16	3.1
**Annual Income**		
Less than $15,000	153	29.9
$15,0010 – $29,000	127	24.8
$30,000 – $49,000	180	35.2
$50,000 and over	52	10.2
**Stay in Canada**		
Less than 1 year	60	11.7
1–5 years	93	18.2
6–10 years	64	12.5
10 years and over	295	57.6

**Table 2:** Change In View Towards TRM (n = 512)

	**Change in View**		
	**Yes**	**No**	**Total**

**Place of Birth (x^2 ^= 0.079, p < 0.779)**			
Ghana	123 (27.3%)	327 (72.7%)	450 (100%)
Canada	18 (29.0%)	44 (71.0%)	62 (100%)
**Gender (x^2 ^= 0.798, p < 0.372)**			
Male	74 (26.0%)	211 (74.0%)	285 (100%)
Female	67 (29.5%)	160 (70.5%)	227 (100%)
**Marital Status (x^2 ^= 1.141, p < 0.285)**			
Married	77 (29.6%)	183 (70.4%)	260 (100%)
Single	64 (25.4%)	188 (74.9%)	252 (100%)
**Age (x^2 ^= 0.049, p < 0.824)**			
Under 40 years	68 (27.1%)	183 (72.9%)	251 (100%)
40 years and over	73 (28.0%)	188 (72.0%)	261 (100%)
**Highest Educational Level (x^2 ^= 7.179, p < 0.007)**			
Secondary & below	81 (33.1%)	164 (66.9%)	245 (100%)
Post-secondary	60 (22.5%)	207 (77.5%)	267 (100%)
**Religion (x^2 ^= 10.894, p < 0.012)**			
Christian	84 (26.5%)	233 (73.5%)	317 (100%)
Traditionalist	23 (24.5%)	71 (75.5%)	94 (100%)
Free Thinker	24 (46.2%)	28 (53.8%)	52 (100%)
Muslim	10 (10.4%)	39 (79.6%)	49 (100%)
**Ethnic Group (x^2 ^= 33.702, p < 0.000)**			
Akan	48 (21.3%)	177 (78%)	225 (100%)
Ga	27 (37.5%)	45 (62.5%)	72 (100%)
Ewe	17 (25.8%)	49 (74.2%)	66 (100%)
Hausa	25 (47.8%)	28 (52.8%)	53 (100%)
Frafra	11 (24.4%)	34 (75.6%)	45 (100%)
Mamprusi	2 (5.7%)	33 (94.3%)	35 (100%)
Dagomba	9 (56.3%)	7 (43.8%)	16 (100%)
**Self-Reported Annual Income (x^2 ^= 5.584, p < 0.018)**			
$29,000 or less	89 (31.8%)	191 (68.2%)	280 (100%)
$30,000 or more	52 (22.4%0	180 (77.6%)	232 (100%)
**Length of Stay in Canada (x^2 ^= 10.571, p < 0.001)**			
10 years and under	76 (35.0%)	141 (65.0%)	217 (100%)
Over 10 years	65 (22.0%)	230 (78.0%)	295 (100%)

### Changes in views towards TRM

From Table 2, 27.3% of those who were born in Ghana reported that they had changed their views on TRM as a result of their stay in Canada. Of those who were born in Canada, nearly 30% reported that they had changed their views on TRM. There is no significant relationship between place of birth and change in attitude toward TRM. Despite the fact that Ghanaians in GTA have generally positive attitudes towards TRM, there are also some concerns about its preparation, storage and preservation. For instance, there were concerns about the unavailability of 'expiry dates' on the medicines produced by the traditional healers, and the unhygienic conditions under which some of these medications are prepared:

"Honestly, the only change in my attitude toward TRM is the unavailability of expiring dates on the medications. When in Ghana, I was not very much concerned about it because most of TRM in Ghana are freshly prepared, but in Canada we normally have to rely on already prepared or mixed herbs from Ghana and I consider this to be dangerous.... I am now aware that even prescription drugs have expiring dates. If the consumption of expired modern medicine is dangerous, then you can imagine how dangerous expired TRM will be" (25 year-old male Akan, Group 1).

Some respondents also indicated that traditional healers may overstate their claim on the efficacy of their medications:

"The problem with traditional healers is that some of them claim more than they can cure and there are a lot of unqualified people practicing out there, especially at the local lorry parks where all sorts of herbs and mixtures are sold to innocent people" (51 year-old male free thinker, Group 2).

A majority of Ghanaians have resorted to the use of 'faith healers' as part of their health seeking behaviour in the GTA. Table 2 shows the cross-tabulation of demographic characteristics and change in view toward TRM. From the focus group discussions, a majority indicated that they have not changed their general attitude towards TRM despite their exposure to modern medicine and technology:

"Although, I see better medicine and modern technology in Canada, my views on TRM has not changed at all...Err.. I consider Traditional medicine to be very powerful and effective than the Whiteman's medicine in the treatment of diseases but now I know that some of the traditional healers have been claiming too much (45 year-old female, Group 1).

This view is consistent with the quantitative data where 72.5% answered 'NO' to the question: "Do you think that your views on TRM as practiced in Ghana have changed as a result of your staying in Canada?" (see Table 2). Some had concerns regarding the preparation and storage of TRM and training of traditional healers. For instance, there were concerns about the hygienic nature of the medicine:

"Yes, my views on the hygienic nature of the preparation of TRM have changed. I feel that in most cases, the healers do not prepare the medicines under hygienic conditions. I used to overlook this when I was in Ghana, but now some TRM do not seem attractive to me as a result of these concerns" (42 year-old male, Ga, Group 1).

There were no significant differences between Ghanaian immigrants who had lived longer in Canada and those who have lived shorter in Canada as far as their attitudes towards TRM was concerned (Table 2). During the focus group discussions, there was no difference between the views of those who have lived longer in Canada and those who have lived shorter in Canada in their terms of their views on TRM. The following statement captures the views of a participant who has lived in Ghana for nearly twenty years:

"I came to Canada in 1983 and within this period, l have visited Ghana on four occasions and anytime that l go to Ghana, l bring some herbal medicine that l cannot find here in case l will need them. Right now, I have some of them in my cupboard and this shows that despite the availability of modern medicine here, l prefer TRM" (36 year-old male Muslim, Group 1).

From Table 2, we can see that 33.1% of those with educational levels up to the secondary level reported a change in attitude towards TRM as a result of their staying in Canada. Of 12 those who had post-secondary education, 22.5% reported a change. There may be a causal relationship between educational level and a change in attitude towards TRM as a result of staying in Canada. Ghanaians with higher levels of education are less likely to report a change in attitude toward TRM. It has been documented that the level of a Ghanaians' education coupled with exposure to the Western World (where medicalization is rule rather exception), has had an influence on their views and opinions about TRM [8], although their cultural beliefs might still be intact; [1] the more educated Ghanaians are, the more likely they will be sceptical toward TRM. In our research, there was a significant relationship between educational level and change in attitude toward TRM. Those whose educational level was below the secondary school level were more likely to view TRM negatively. However, a subtler notion appeared the focus group discussions, where participants with university level education (acquired in Ghana and/or Canada) had mixed feelings about TRM. The following contradictory statements were made by university educated Ghanaians. Some saw downsides to TRM:

"I have lived in the village before and in fact I like living in the village and so I know how they prepare TRM. Sometimes within dirty things...for instance dirty dishes or calabash, they won't wash it, they pour portion upon portion into it and so because of that it is really a concern and we should improve the potency for us. Maybe, the medicine may have power to do great work, but because of dirt and other things that we add, it reduces the potency. Secondly, when you buy the white man's medicine, he tells you that 'in the morning, take one, in the evening, take one' and as for our people's medicine, there is no dosage. For the '*alafia bitter'*, people can drink as much as they want. But if they can specify that you take a spoonful every morning or evening it will go a long way to help" (37 year-old male, university graduate, Group 3).

And, others had good experience of TRM,

"As I said, for me, I used to have severe asthma and I could not do anything with my cousins. They say, in Fante-land, there is somebody there with bitter medicine, but if you are able to drink it, you will vomit all the time, you will vomit and only phlegm will come out and if you finish vomiting, you will be okay and it will not come again... For ten years now, I have not got any asthma, from ten years now, I have not got asthma at all" (33 year-old female Christian, Group 3).

Thus, the focus group discussions did not support the view that more educated Ghanaians are more likely to change their view of TRM.

Table 2 shows that 31.8% of those who earn $29,000.00 or less reported in attitude towards TRM as a result of staying in Canada. Of those who earn $30,000.00 or more, 24.4% reported a change in attitude. There may be a causal relationship between income level and a change in attitude toward TRM as a result of staying in Canada. Ghanaians who earn $29,000.00 or less are more likely to change their attitudes toward TRM.

There was no statistically significant difference between change in attitude towards TRM and the gender of the respondents (see Table 2), which is not consistent with previous findings [1,13,26,46]. However, the gender of the respondents had no influence on the way they thought about TRM as indicated elsewhere [34]. The results of this research is not surprising in view of the fact that it may be conceivable that the females may have discussed their 'public' views on TRM as opposed to their 'private' views which may not be appropriate as far as socially constructed ideas of health and illness are concerned [28]. The responses of the females may also be partly due to "the pressure associated with the socialisation of women into the gender-specific roles which the Ghanaian society expects from them [44]."

## Discussion

Being born in either Canada or Ghana does not have any significant effect on the attitudes and opinions of Ghanaians toward TRM. They prefer traditional healers who can also take care of their spiritual needs, as noted by previous researchers [27,35,36]. The majority of Ghanaians in Canada have adopted a mix-and-match approach of health seeking behaviour whereby the decision to use either TRM or modern medicine is based on the nature of the health problem and the perceived success rate of TRM. If the ultimate aim of acculturation involves a process of adaptation in which Ghanaians alter their attitudes and behaviours to more closely resemble those of Canadians[23], then Ghanaians in the GTA who have not fully acculturated into the Canadian society [11. According to Loue, groups and individuals that do not acculturate are more likely to rely on alternative models of health and illness leading to increase in the utilization of TRM [27].

It is interesting to note that the reasons given by Ghanaians for any changes in their attitude toward TRM was negative (i.e. away from believing in it once landed in Canada). However, it is conceivable that people who were skeptical whilst in Ghana, immigrated to Canada, and had some poor experiences with Western medicine and subsequently became more positive about the usefulness of TRM, for example:

"Here, I can see that there are a lot of children with asthma who have been given medication, but yet still the disease is still there. I have not taken the one here and so I do not know the difference apart from the TRM that I took..." (33 year-old female Christian, Group 3).

Lee and colleagues also found a similar experience among the Chinese in the GTA. The respondents also indicated that they have not seen any changes in the attitudes of other Ghanaians towards TRM as a result of their staying in Canada [20]. This is consistent with the quantitative data where 72.9% observed no change in attitude. This is not consistent with the prediction that the beliefs in, and practice of ethno-medicine will become a thing of the past with the increase in the adoption of modern life [1].

### The importance of the spiritual element of TRM

The majority of Ghanaians in the GTA seem to have a preference for healers who can address the spiritual in parallel with the physical health. This is consistent with the increase in 'spiritual' or 'faith healing' churches in the GTA. These healers are said to have a 'third eye' capable of detecting both spiritual and physical health problems [8]. However, there is a sense of having to conform and keep TRM usage secret among Ghanaians who are well educated and/or Christians [1,8,37]. This theme was repeated in this research:

"It could be true that most of the people use the services of the herbalists and not fetish-priests, but on the other hand, I believe very well that some of them visit gods and fetish-priests but they do not say the truth because where we are now, it is something like everybody is worshipping God and so you don't want to identify yourself within these people that if you use fetish-priests or other gods. Normally, people do not say the truth in surveys because he knows that if he tells you the truth, Kofi will know that I also go to the fetish-priests" (37 year-old male traditionalist, Group 3).

Based on the above observation, it is conceivable that some participants may not have been truthful about their usage of certain aspects of TRM during the focus group discussions. Some of the things they said 'publicly' during the focus group discussions may be different from their 'private' views [38]. Although there are changing perceptions of the causes of diseases among Ghanaians, most Ghanaians in the GTA tend to operate within the 'Personalistic Medical System' [39]. Depending on the circumstances in which the disease occurs, the disease may be classified as being supernaturally or naturally caused [43,44]. The 'Personalistic Medical System' applies in situations where community members believe that punishment or aggression directed at a person or a patient is due to the deliberate interventions of agents who may be supernatural (gods, spirits or ancestors) or human beings with evil powers.

### Mix and match approach to health care

An interesting issue that came out of this research was the mix-and-match approach to health seeking behaviour whereby Ghanaians choose between either TRM or modern medicine depending on the nature of illness or health problem. For example, our questionnaire survey found TRM was most commonly used for the treatment of leg injuries, as have previous studies on TRM amongst Ghanaians [1,35]. However, the decision to use TRM depended on cost, type of illness and the perceived success rate of TRM in treating that particular illness or health problem [43,44]. There was also a perception amongst the several focus group participants in that some TRM practitioners claim too much [8,43,44]. The general feeling was that TRM is not for everybody, for example some people may have negative reactions to it. However, it is not surprising that some TRM is said to be capable of treating a variety of illnesses or diseases since modern medicine such as aspirin is also given for twenty-odd different symptoms or problems. Some Ghanaians are comfortable with 'modernity' (Western, industrialized, civilized, etc) during the acculturation process in Canada. To them being 'modern' is better than being 'traditional' and they may agree with the stand that acculturation everywhere among all peoples is towards being 'modern' [24].

The changes in some Ghanaians' cultural identity indicated by the 37 year-old male Christian are consistent with the concept of 'plasticity of modernity' [42]. Thus, some Ghanaians have changed some of their traditions, practices and customs to adapt to Western civilization. Such Ghanaians are not longer willing to conform to TRM for the sake of West African Traditional Religion.

Despite elements of separation and marginalization in the health seeking behaviour of Ghanaians in Canada, it appears that a majority of Ghanaians are pursuing integration and assimilation in the acculturation process in Canada more in line with Reece and Palmgreen's notion of "stress-adaptation-growth process" [30]. According this theory, individuals acculturating in a new country are "forced to broaden their perspective to include the parameters of both cultures" as a survival strategy [30]. In the course of such integration and assimilation processes, Ghanaians give up or modify some of their attitudes towards TRM to embrace the 'modern' or 'civilized' way of health seeking behaviour dominant in Canada.

Although 'modernity' is said to be founded on "ruthless undermining of tradition" [33], there is no evidence in this research to suggest that West African Traditional Religion has been undermined to such an extent that there is a change in attitude and opinion of Ghanaians in the GTA towards TRM.

### Implications for TRM Practice, Health Care Policy and Research in Canada

Although this paper did not explicitly investigate Ghanaians' satisfaction with the current Canadian health care system, there is enough evidence to suggest that Canadian immigrants are not receiving culturally-appropriate or culturally-sensitive health care services [47,19]. Therefore, this paper is advocating a Multicultural health care system in Canada. Multicultural health care is a "health care which is cultural, racially and linguistically sensitive and responsive [48]."

The evidence in this paper indicates that Ghanaians have adopted a pick-and-mix approach by utilizing the services of both TRM and modern medicine practitioners depending on the nature of the health problem. Therefore, this paper recommends (1) a policy of parallel development of multiple health systems in the short run; and (2) ultimately, a policy of active collaboration between fully recognized health systems. Under the former health system, both TRM and modern medicine practitioners are officially recognized and they serve their patients through separate but equal systems [49]. Under the latter health care system, there is an assumption that there is equity, mutual respect and understanding among TRM practitioners and doctors [50].

This paper raises questions that need to be addressed in further research on TRM in Canada given the attitudes and opinions of Ghanaians who have had the opportunity of experiencing the advantages and disadvantages of both traditional and Western medical systems on a much larger scale. For instance, further research should be conducted to determine under what conditions Ghanaians would use TRM if it became part of the Canadian health care delivery system. It has been suggested that:

"Fieldwork needs to be done at the community level to arrive at a better understanding and assessment of the community's opinion concerning a possible role of traditional medicine in basic health care [51]."

In a plea for a community perspective on the integration of TRM and modern medicine, Van der Geest observed that local communities do not expect any improvement in basic health care when TRM practitioners are integrated into the system [51].

## Conclusion

Ghanaians in the GTA have been pursuing 'integration' and 'assimilation' in their acculturation in Canada. Some have given up or modified some of their attitudes and opinions toward TRM to embrace the 'modern' or 'civilized' way of living. Although modernity is said to be founded on the "ruthless undermining of tradition [45]," there is no evidence to suggest that Ghanaian traditional religion has been undermined to such an extent that there is a major change in attitudes towards TRM.

## Competing interests

The authors declare that they have no competing interests.

## Authors' contributions

KBB conceived of the study and both authors contributed to its design. KBB collected the data and conducted most of the analysis. KBB and ERvT contributed equally to the writing of the manuscript and both approved the final version of the paper.

## Pre-publication history

The pre-publication history for this paper can be accessed here:


